# The Hh pathway promotes cell apoptosis through Ci-Rdx-Diap1 axis

**DOI:** 10.1038/s41420-021-00653-3

**Published:** 2021-09-24

**Authors:** Bin Liu, Yan Ding, Bing Sun, Qingxin Liu, Zizhang Zhou, Meixiao Zhan

**Affiliations:** 1 College of Life Sciences, Shandong Agricultural University, Tai’an, China; 2 Department of Anorectum, the First affiliated Hospital of Shandong First Medical University, Ji’nan, China; 3 Zhuhai Interventional Medical Center, Zhuhai Precision Medical Center, Zhuhai People’s Hospital, Zhuhai Hospital Affiliated with Jinan University, Zhuhai, China

**Keywords:** Cell signalling, Apoptosis

## Abstract

Apoptosis is a strictly coordinated process to eliminate superfluous or damaged cells, and its deregulation leads to birth defects and various human diseases. The regulatory mechanism underlying apoptosis still remains incompletely understood. To identify novel components in apoptosis, we carry out a modifier screen and find that the Hh pathway aggravates Hid-induced apoptosis. In addition, we reveal that the Hh pathway triggers apoptosis through its transcriptional target gene *rdx*, which encodes an E3 ubiquitin ligase. Rdx physically binds Diap1 to promote its K63-linked polyubiquitination, culminating in attenuating Diap1−Dronc interaction without affecting Diap1 stability. Taken together, our findings unexpectedly uncover the oncogenic Hh pathway is able to promote apoptosis through Ci-Rdx-Diap1 module, raising a concern to choose Hh pathway inhibitors as anti-tumor drugs.

## Introduction

Multicellular organisms keep homeostasis through a balance between cell proliferation and cell apoptosis. In embryonic development, apoptosis removes unnecessary cells to coordinate organogenesis. In adult tissues, apoptosis could eliminate senescent cells to maintain homeostasis. When the organism undergoes external stimuli, including ultraviolet light and reactive oxygen species, damaged cells are also cleared by apoptosis [1]. Therefore, apoptosis plays important role in both physiological and pathological conditions. Recently, increasing studies have shown that abnormal regulation of apoptosis leads to a variety of human diseases, such as tumors [2] and neurodegenerative diseases [3]. Exploring the mechanism underlying apoptosis is helpful to discover novel drug targets for the treatment of apoptosis-related diseases.

An evolutionarily conserved process during apoptosis is the sequential activation of several caspases, which trigger apoptotic cell death by cleaving many structural and regulatory proteins [4]. As a matter of fact, the activation of caspase cascade is tightly monitored due to a family of anti-apoptotic proteins, termed inhibitor of apoptosis proteins (IAPs) [5]. IAPs are first identified as baculoviral proteins that block the defensive apoptosis of insect cells after infection [6]. In addition, many IAPs comprise a carboxy-terminal RING domain and function as E3 ubiquitin ligases to ubiquitinate pro-apoptotic proteins, including caspases [7]. In fruit flies, *Drosophila* IAP-1 (Diap1) prevents cells from apoptosis through ubiquitinating and subsequent destabilizing *Drosophila* Nedd2-like caspase (Dronc), the initiator caspase ortholog to human Caspase-9 [8, 9]. In cells that undergo apoptosis, the anti-apoptotic activity of Diap1 is suppressed by upstream antagonists, including head involution defective (Hid), Reaper (Rpr), and Grim [5]. These three proteins negatively regulate Diap1 through distinct mechanisms, either by decreasing Diap1 level or by disrupting Diap1−Dronc interaction [10, 11]. Furthermore, Diap1 protein could be degraded by N-end rule pathway [12]. The E3 ligase Ubr3 enhances Diap1 activity though promoting Diap1-Dronc association, without affecting the ubiquitination of Diap1 [13]. In conclusion, Diap1 is a key modulator for cell death, and its activity should be strictly controlled by multiple mechanisms to avoid unfitted apoptosis.

The evolutionarily conserved Hedgehog (Hh) pathway plays important roles in physiological and pathological processes, such as embryogenesis, cell fate determination, tissue damage repair, stem cell maintenance, and tumorigenesis [14]. Inactivation of the Hh pathway leads to developmental defect, while its hyperactivation causes several human cancers [15, 16]. The *Drosophila hh* gene encodes a diffusible ligand, which activates the pathway through binding its receptor Patched (Ptc) with the assist of co-receptors including Ihog/Boi [17, 18]. Ptc inhibits the cell surface accumulation and subsequent activation of Smoothened (Smo), an indispensable transducer for the Hh pathway [19]. Hh ligand is able to bind Ptc to relieve its inhibitory effect on Smo possibly through Smo phosphorylation and deubiquitination, culminating in the Hh pathway activation [20, 21]. During Hh signaling transduction, the transcriptional factor Cubitus interruptus (Ci) is a critical executor [22]. In the absence of Hh ligand, Ci is sequestered in the cytoplasm by the microtubule-associated protein Costal2 (Cos2) with the assist of the scaffold Rack1 [23]. In the presence of Hh ligand, Ci dissociates from Ci-Rack1-Cos2 complex and enters the nucleus to turn on the expression target genes [23]. Among Ci target genes, *roadkill* (*rdx*) encodes an E3 ligase to promote Cullin3 (Cul3)-mediated protein ubiquitination [24]. To date, several studies have shown that the Hh pathway is able to inhibit cell death via upregulating the anti-apoptotic gene *Bcl2* in human tumor cells, providing Hh pathway inhibitors as proapoptotic drugs for tumor treatment [25, 26]. Although *Drosophila* genome encodes two orthologs of *Bcl2*, *buffy* and *debcl*, they do not play an obvious role in apoptosis [27]. Thus, it is still unclear whether and how the Hh pathway regulates apoptosis in *Drosophila*.

To find novel regulators in *Drosophila* apoptosis, we carried out a genetic screen and identified the Hh pathway as a positive regulator of apoptosis. Knockdown of *ci* effectively suppressed Hid-induced apoptosis and small eyes, while overexpression of *ci* or its upstream *smo* showed opposite results. Moreover, Ci aggravated Hid-induced apoptosis through its transcriptional target gene *rdx*, since the loss of *rdx* phenocopied *ci* knockdown. Biochemical analyses revealed that Rdx interacted with Diap1 through its N-terminal MATH domain. We also identified two matched recognition motifs in Diap1 responsible for binding Rdx. Interestingly, Rdx was unable to affect Diap1 protein stability. Furthermore, we found that Rdx promoted K63-linked polyubiquitination on Diap1, and decreased Diap1−Dronc interaction, culminating in inhibition of Diap1 activity. Taken together, our study uncovered an unexpected role of the Hh pathway in apoptosis, and raised a concern to choose Hh pathway inhibitors as anti-tumor drugs.

## Results

### The Hh pathway is a positive regulator for Hid-induce apoptosis

**Fig. 1:**
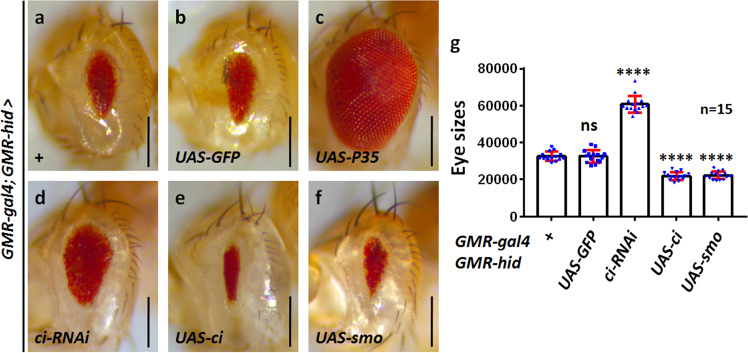
The Hh pathway decreases *GMR*-*hid* eye size. All eyes are oriented anterior left, dorsal up. **a** Overexpression of *hid* using *GM*R promoter leads to the small eye. Compared with the control eye (**b**), P35 rescued the *GMR*-*hid* eye to the normal size (**c**). **d** Silence of *ci* increased *GMR*-*hid* eye. Overexpression of *ci* (**e**) or *smo* (**f**) decreased *GMR*-*hid* eyes. **g** Quantification analyses of **a**–**f** eye sizes (*n* = 15). Scale bars: 200 μm for all eyes.

**Fig. 2:**
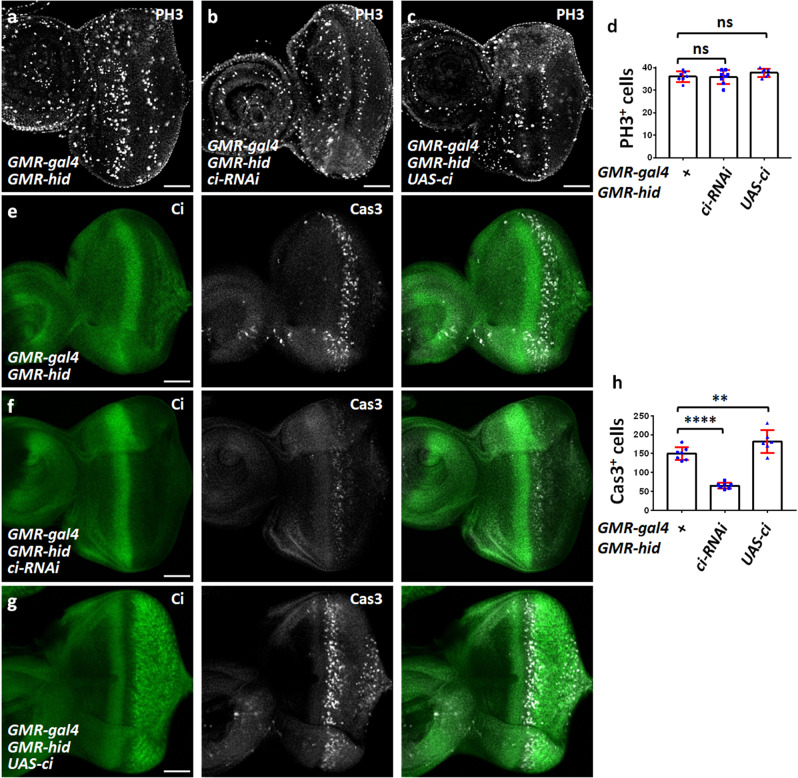
Ci promotes Hid-induced apoptosis. All eye imaginal disks shown in this figure were oriented with anterior on the left. **a** A control eye disc was stained with PH3 antibody to mark proliferative cells. **b** Knockdown of *ci* did not affect PH3 signals under *GMR*-*hid* background. **c** Overexpression of *ci* did not regulate cell proliferation. **d** Quantification analyses the PH3-positive cells of **a**–**c** (*n* ≥ 6). **e** A control eye disc was stained to show Ci (green) and Cas3 (white). **f** Knockdown of *ci* decreased apoptosis. **g** Overexpression of *ci* promoted apoptosis. **h** Quantification analyses the Cas3-positive cells of **e**–**g** (*n* ≥ 6). Scale bars: 50 μm for all eye disks.

To explore novel regulators of apoptosis, we established a modifier screening, in which the pro-apoptotic gene *hid* was overexpressed in *Drosophila* eyes using the eye-specific *glass multimer repeat* (*GMR*) promoter to induce massive cell death. Small eyes of *GMR*-*hid* (Fig. 1a) provided a sensitive background for subsequent screening, since partially rescued eyes are readily noticeable. Compared to the control (Fig. 1b), ectopic expression of the well-known anti-apoptotic baculovirus P35 protein almost restored the eye of *GMR*-*hid* to wild-type size (Fig. 1c), suggesting that the small eye of *GMR*-*hid* was indeed caused by excessive apoptosis. Next, we expressed transgenic RNAi lines to identify suppressors of the small eye. From this screening, we found that knockdown of *ci* apparently increased the eye size (Fig. 1d, g). In contrast, overexpression of *ci* decreased the eye size under *GMR*-*hid* background (Fig. 1e, g). Given that Ci is the unique transcriptional factor of the Hh pathway, we wanted to examine whether the pathway is involved in modulating Hid-induced apoptosis. Similar to Ci, overexpression of the upstream component Smo also reduced *GMR*-*hid* eyes (Fig. 1f, g). Since the Hh pathway regulates cell proliferation in *Drosophila* [28], we sought to test whether Ci controls *GMR*-*hid* eye size through cell proliferation. Compared with the control disc (Fig. 2a, d), neither *ci* knockdown (Fig. 2b, d) nor *ci* overexpression (Fig. 2c, d) influenced the level of phosphor-histone H3 (PH3), which is a marker for cell division [29]. In contrast, knockdown of *ci* decreased (Fig. 2e, f, h), while overexpression of *ci* elevated apoptosis under GMR-hid background (Fig. 2e, g, h), suggesting that the Hh pathway promotes Hid-induced cell death.

### The Hh pathway promotes apoptosis through Rdx

**Fig. 3:**
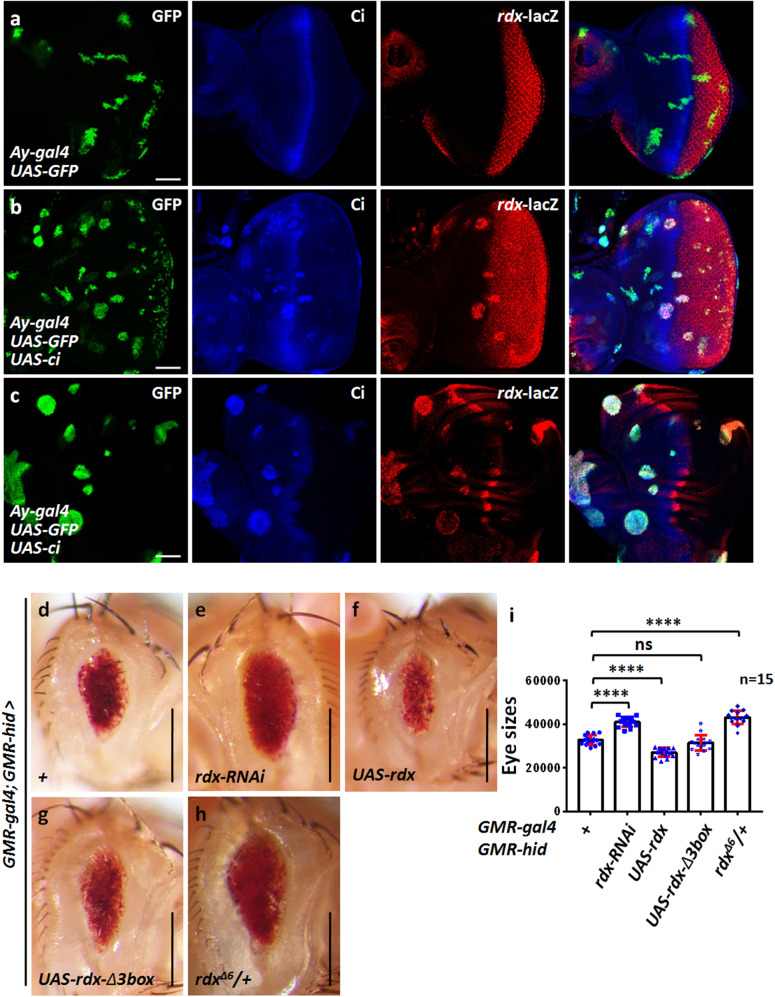
The Hh pathway regulates apoptosis through Rdx. **a** A control eye disc was stained to show GFP (green), Ci (blue), and *rdx*-lacZ (red). Of note, *rdx*-lacZ expresses in the posterior region of the eye disc. **b** Overexpression of *ci* activated *rdx*-lacZ expression in the eye disc. **c** Ci was able to turn on *rdx*-lacZ expression in the wing disc. Compared to the control eye (**d**), knockdown of *rdx* elevated *GMR*-*hid* eye (**e**), while overexpression of *rdx* decreased *GMR*-*hid* eye (**f**). **g** Overexpression of *rdx-Δ3box* failed to affect *GMR*-*hid* eye. **h** Deletion one copy of endogenous *rdx* increased *GMR*-*hid* eye. **i** Quantification analyses of **d**–**h** eye sizes (*n* = 15). Scale bars: 50 μm for all disks and 200 μm for all adult eyes.

The Hh pathway exerts biological function through its transcriptional targets. Well-documented target genes of Hh signaling include *knot* (*kn*) *decapentaplegic* (*dpp*), *patched* (*ptc*), *engrailed* (*en*) and *roadkill* (*rdx*) [30, 31]. Our previous study has demonstrated that overexpression of *rdx* produces shriveled wings, possibly due to apoptosis [32]. Thus, we focused on rdx in following studies. First, we employed a *rdx*-lacZ reporter [33], which the lacZ coding sequence was inserted downstream of *rdx* promoter, to monitor *rdx* expression. Compared with the control eye disc (Fig. 3a), overexpression of *ci* substantially increased *rdx*-lacZ expression (Fig. 3b). We further showed that Ci also activated *rdx*-lacZ expression in the wing disc (Fig. 3c), suggesting that *rdx* is a *bona fide* transcriptional target of Ci.

To test whether Rdx is involved in regulating Hid-induced apoptosis, we modulated Rdx level under *GMR*-*hid* background and analyzed eye sizes. Compared with *GMR*-*hid* control (Fig. 3d, i), knockdown of *rdx* enlarged the eye (Fig. 3e, i), whereas *rdx* overexpression reduced the eye (Fig. 3f, i). In addition, we used a null allele of *rdx*, *rdx*^*Δ6*^ [33], to validate the result. Similiar to *rdx* RNAi, The eye of *GMR*-*hid* was increased in *rdx*^*Δ6*^ heterozygote background (Fig. 3h, i). We could not delete two copies of *rdx*, due to *rdx*^*Δ6*^ homozygote was embryonic lethal [33]. Rdx is a Cul3-based E3 ligase, which recruits substrates to Cul3 for ubiquitination [34]. Cul3 acts as a scaffold to bridge E2 ubiquitin-conjugating enzymes and E3 ligases [35]. Rdx protein contains a 3box domain responsible for its interaction with Cul3 [32]. Deletion of 3box domain abolishes Rdx-Cul3 association and its E3 ligase activity [32]. We found that Rdx-Δ3box failed to decrease the eye size of *GMR*-*hid* (Fig. 3g, i), indicating Rdx E3 ligase activity is indispensable for its regulation on apoptosis. Taken together, these findings suggest that the Hh promotes Hid-induced apoptosis through Rdx.

### Rdx interacts with Diap1

**Fig. 4:**
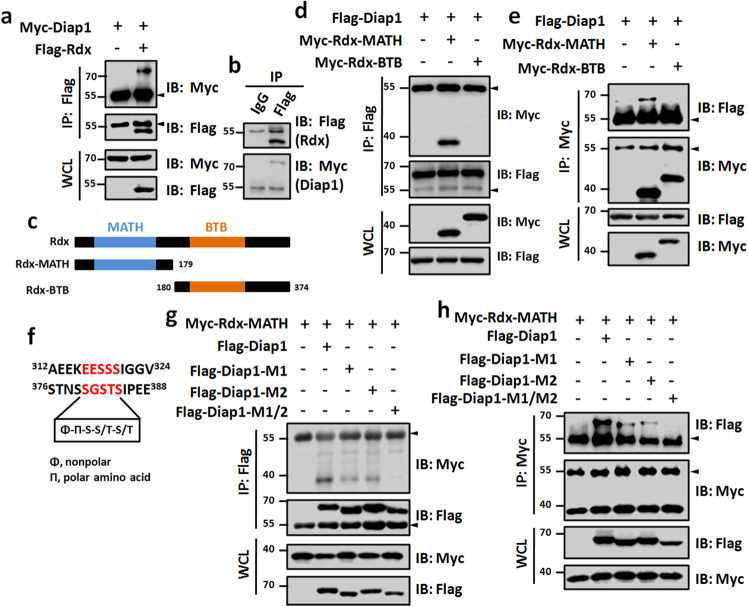
Rdx binds Diap1. **a** Flag-tagged Rdx protein could pull down Myc-tagged Diap1 protein in 293 T cells. **b** 293 T cells transfected with Myc-Diap1 and Flag-Rdx were divided into two parts to carry out IP using mouse IgG or mouse anti-Flag antibody. The following WB results showed that only mouse anti-Flag antibody was able to pull down Flag-Rdx and Myc-Diap1. **c** The schematic drawing shows the domains in Rdx protein and the truncated fragments used in subsequent co-IP assays. **d** Flag-tagged Diap1 protein pulled down Rdx-MATH protein, but not Rdx-BTB protein in 293 T cells. **e** Myc-tagged Rdx-MATH interacted with Flag-Diap1. **f** Diap1 protein contained two SBCs. **g**, **h** Reciprocal co-IP experiments showed mutation of SBCs abolished Diap1 interaction with Rdx-MATH. Above all, arrowheads indicate heavy IgG, and WCL represents whole cell lysate.

Previous studies have clearly elucidated that Hid induces apoptosis through inhibiting Diap1 [36], and Diap1 overexpression could totally restore *GMR*-*hid* to wild-type eye size [37], suggesting that Diap1 is important for Hid-caused apoptosis. Since our above results showed Rdx promotes apoptosis in an E3 ligase-dependent manner, we speculated that Rdx binds Diap1 to accelerate its ubiquitination and proteasome-mediated degradation. The co-immunoprecipitation (co-IP) assay indeed showed the interaction between Rdx and Diap1 (Fig. 4a, b). Rdx is comprised of a MATH domain in its N-terminus and a BTB domain in its C-terminus (Fig. 4c). To determine which domain on Rdx is responsible for its interaction with Diap1, we generated two truncated forms of Rdx, which exclusively contained MATH or BTB domain (Fig. 4c). The co-IP assay showed that Flag-tagged Diap1 protein only pulled down Rdx-MATH (Fig. 4d). Reciprocally, Rdx-MATH, not Rdx-BTB interacted with Diap1 (Fig. 4e), together suggesting that Rdx binds Diap1 through its N-terminal MATH domain.

The previous study has demonstrated that Speckle Type POZ Protein (SPOP), the mammalian counterpart of Rdx, recognizes a conserved degron named as SPOP binding consensus (SBC) [38]. Through examining the protein sequence of Diap1, we found two putative SBCs: EESSS (termed degron 1) and SGSTS (termed degron 2) (Fig. 4f). To test which SBC is required for Diap1-Rdx interaction, we constructed three Diap1 mutants with the replacement of each or both degrons by AAAAA. Mutation of degron 1 (M1) or degron 2 (M2) effectively diminished the interaction between Diap1 and Rdx-MATH, while mutation of both degrons (M1/2) totally abolished this interaction (Fig. 4g, h). These results demonstrate that Rdx binds two SBCs on Diap1 protein through its N-terminal MATH domain.

### Rdx does not affect the stability of Diap1 protein

**Fig. 5:**
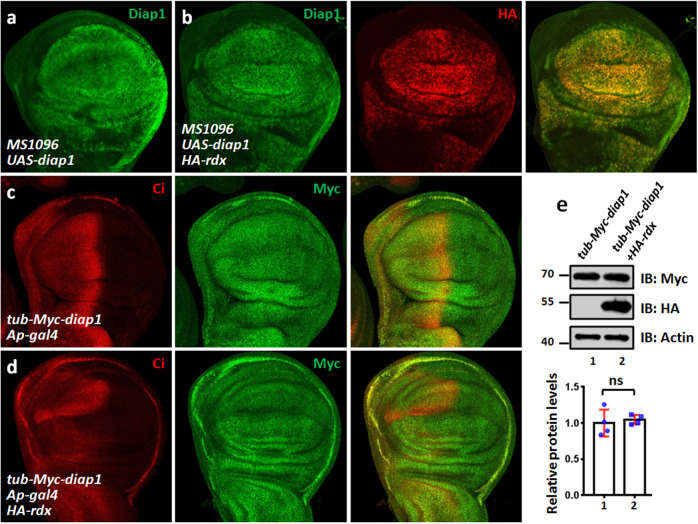
Rdx fails to destabilize Diap1. **a** A wing disc expressing *diap1* by *MS1096* was stained with Diap1 antibody. **b** A wing disc simultaneous expressing *diap1* and HA-*rdx* was stained to show Diap1 (green) and HA (red). Of note, Rdx did not decrease Diap1 protein. **c** A control wing disc from *tub*-Myc-*diap1* was stained to show Ci (red) and Myc (green) antibodies. *tubulin* promoter drives Myc-*diap1* wide expression in the wing disc. **d** A wing disc expressing *rdx* by *Ap-gal4* under *tub*-Myc-*diap1* background was stained with Ci (red) and Myc (green) antibodies. Overexpression of *rdx* did not affect Myc-tagged Diap1 protein. **e** IB assays of lysates from control wing disks or wing disks expressing HA-*rdx* with *nub-gal4* driver. Approximately 50 wing disks were dissected, lysed, and blotted with indicated antibodies. Actin acts as a loading control. Relative densities of Myc-Diap1 bands were shown below. Data were presented as means ± SD from four independent experiments.

Since the above studies reveal that the E3 ligase Rdx binds Diap1, it is necessary to test whether Rdx promotes Diap1 degradation. Compared with *diap1* expression alone (Fig. 5a), co-expression of *rdx* was unable to decrease Diap1 protein in the wing disc (Fig. 5b). To validate this result, we generated a *tub*-Myc-*diap1* transgenic fly, which drives Myc-tagged *diap1* expression using *tubulin* promoter. Immunostaining with anti-Myc antibody showed that *tub*-Myc-*diap1* widely expressed in the wing disc (Fig. 5c), consistent with the expression pattern of *tubulin* promoter. Overexpression of *rdx* apparently decreased its well-known substrate Ci, but without affecting Myc-Diap1 level (Fig. 5d). Furthermore, we extracted protein of wing disks for western blot (WB) assay, and also revealed that overexpression of *rdx* did not change Myc-Diap1 level (Fig. 5e). Overall, the results together suggest that Rdx binds Diap1, but does not promote Diap1 degradation.

### Rdx promotes K63-linked ubiquitination of Diap1

**Fig. 6:**
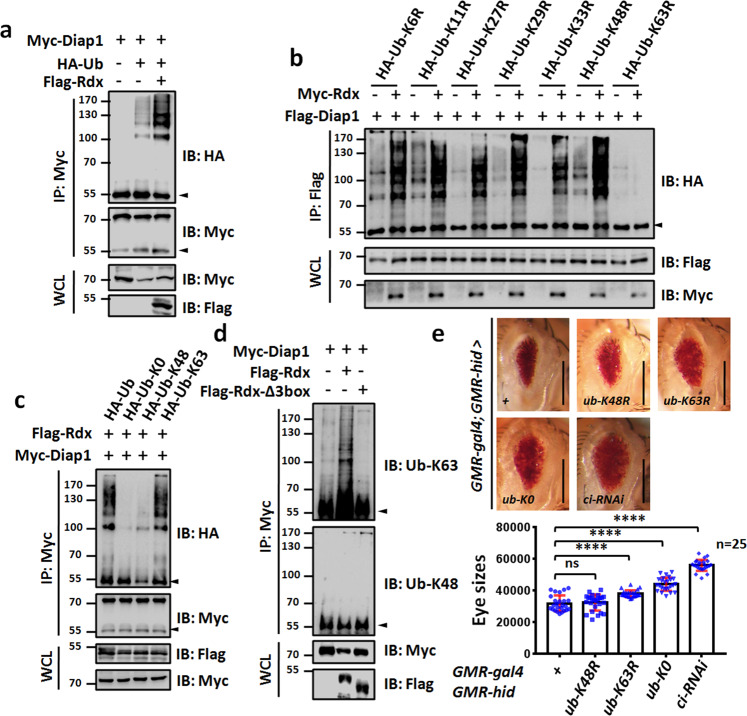
Rdx promotes K63-linked ubiquitination on Diap1 protein. **a** Rdx enhanced ubiquitination of Diap1. **b** Rdx failed to promote Diap1 ubiquitination when HA-Ub-K63R was expressed in 293T cells. Notably, K63 in Ub was important for Rdx-induced Diap1 ubiquitination. **c** Rdx promoted K63-linked polyubiquitination on Diap1. **d** Rdx elevated K63-linked ubiquitination of Diap1 protein, while Rdx-Δ3box failed to do so. **e** Overexpression of *ub-K63R* or *ub-K0* enlarged *GMR*-*hid* eye, while *ub-K48R* did not. *ci*-RNAi acts as a positive control. Quantification analyses of eye sizes (*n* = 25) were shown below. Scale bars: 200 μm for all eyes. Above all, arrowheads indicate heavy IgG, and WCL represents whole cell lysate.

The above results demonstrate that the E3 ligase activity is important for Rdx to promote Hid-induced apoptosis, since deletion of 3box abolishes its function. In addition, Rdx shows specific binding to Diap1. We next sought to determine whether Rdx accelerates ubiquitin modification of Diap1 protein. The cell-based ubiquitination assay revealed that Rdx indeed enhanced Diap1 ubiquitination (Fig. 6a).

For ubiquitination, the first ubiquitin (Ub) is covalently attached to the lysine residue (K) of the substrate [39]. The following Ub is attached to one of the lysine residues of the previous Ub to form polyubiquitin chain [39]. Ub contains seven lysine residues (K6, K11, K27, K29, K33, K48, and K63), which can be used to create inter-Ub linkages during polyubiquitin chain formation [40]. It is well known that substrates with the different linkage of polyubiquitin chains have distinct fates. For instance, K33-linked polyubiquitination is involved in modulating protein trafficking [41]. K48-linked polyubiquitination generally targets proteins for proteasomal degradation [42]. K63-linked polyubiquitin modification usually plays a non-degradative role, instead regulates protein localization and protein−protein interaction [43, 44]. To examine which type of ubiquitination occurs on Diap1 protein, we employed several Ub mutants, in whom one of the seven Ks was substituted by arginine (R). The results showed that Rdx failed to promote Diap1 ubiquitination when Ub-K63R was used (Fig. 6b). On the other hand, Ub-K0, in which all Ks are replaced by Rs, could totally abolished Rdx-induced Diap1 ubiquitination (Fig. 6c). However, Ub-K63, which only harbor one K on 63, had a similar effect as wild-type Ub on Rdx-induced Diap1 ubiquitination (Fig. 6c). To reinforce this result, we chose Ub-K63 and Ub-K48 antibodies to distinguish K63-linked and K48-linked polyubiquitin chains. The co-IP assays showed that Rdx exclusively enhanced Ub-K63 signal, not Ub-K48 signal, suggesting that Rdx promotes K63-linked polyubiquitination on Diap1 protein (Fig. 6d). Besides, Rdx-Δ3box did not influence Diap1 ubiquitination (Fig. 6d), further proving that Rdx increases Diap1 ubiquitination through Rdx-Cul3 E3 ligase.

To investigate the revelance between K63-linked polyubiquitination and Hid-induced apoptosis, we generated three transgenic flies to express Ub-K48R, Ub-K63R, or Ub-K0 respectively. Compared with control eyes, Ub-K63R and Ub-K0 enlarged *GMR*-*hid* eyes, whereas Ub-K48R did not (Fig. 6e), indicating that K63-linked polyubiquitination possibly inhibits Diap1 activity. It is worth noting that although overexpression of Ub-K63R indeed enlarged *GMR*-*hid* eyes, the enlargement was not significant, likely due to endogenous wild-type Ub.

### Rdx represses Diap1−Dronc interaction

**Fig. 7:**
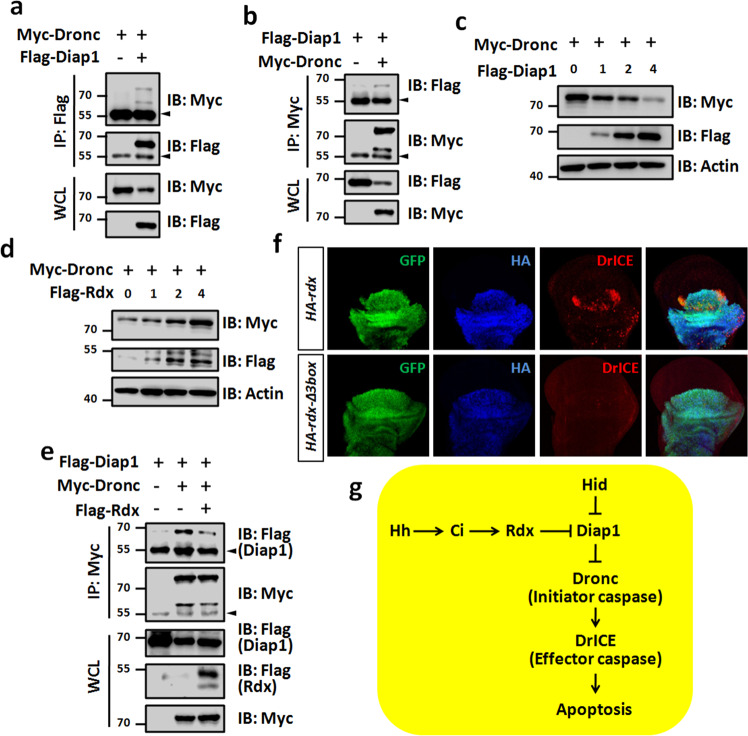
Rdx attenuates the interaction between Diap1 and Dronc. **a** Flag-tagged Diap1 protein pulled down Myc-tagged Dronc protein in 293T cells. **b** Myc-tagged Dronc protein pulled down Flag-tagged Diap1 protein in 293T cells. **c** Diap1 downregulated Dronc in a dose-dependent manner. **d** Rdx elevated Myc-tagged Dronc protein in a dose-dependent manner. **e** Rdx inhibited the interaction of Diap1 and Dronc. **f** Wing disks expressing HA-*rdx* or HA-*rdx-Δ3box* by *Ap-gal4* were stained to show GFP (green), HA (blue), and DrICE (red). Of note, HA-Rdx could trigger apoptosis, whereas HA-Rdx-Δ3box failed to do so. GFP marks the expression region of *Ap-gal4*. **g** A proposed model of the Hh pathway promoting apoptosis. The Hh pathway turns on *rdx* expression through the transcriptional factor Ci. In turn, Rdx binds Diap1 and promotes its K63-linked polyubiquitination to attenuate its association with Dronc, culminating in DrICE activation and apoptosis.

Previous studies have revealed that K63-linked polyubiquitin chains could serve as a scaffold to regulate protein complex formation [45]. We hypothesized that Rdx-mediated K63-linked polyubiquitination likely influence the interaction of Diap1 with Dronc, a critical target of Diap1. Reciprocal co-IP assays showed that an interaction exists between Diap1 and Dronc (Fig. 7a, b). Consistent to previous studies, Diap1 was capable of decreasing Dronc protein in a dose-dependent manner (Fig. 7c). Contrarily, we found that Rdx could stabilize Dronc protein (Fig. 7d). The co-IP results revealed that Rdx attenuated Diap1−Dronc interaction (Fig. 7e). In addition, Rdx was able to trigger apoptosis, while Rdx-Δ3box failed to do so (Fig. 7f). Taken together, these results suggest that the E3 ligase Rdx promotes K63-linked polyubiquitination of Diap1 to attenuate Diap1−Dronc interaction, culminating Dronc stabilization and apoptosis (Fig. 7g).

## Discussion

Apoptosis is a process of programmed cell death that helps to clear away unwanted or dangerous cells. Under normal physiological conditions, apoptosis is maintained at a low level to avoid unfitted cell death. In fact, staining wild-type wing and eye disks with active-caspase3 antibody show weak signals [46, 47]. Therefore, it is challenging to identify negative regulators of apoptosis using RNAi-mediated genetic screening under the normal physiological background. To overcome this difficulty, we developed a modifier screening in this study. Overexpression of the pro-apoptotic *hid* using *GMR* promoter produces small eyes due to excessive apoptosis. We conducted RNAi-mediated screening under *GMR*-*hid* background to identify which RNAi could enlarge the eye size. Through unbiased screening, we found that *ci* RNAi apparently increased *GMR*-*hid* eye. In contrast, overexpression of *ci* or its upstream *smo* decreased the eye size, together suggesting that the Hh pathway is able to promote Hid-induced apoptosis. Next, we showed that the E3 ligase Rdx could mimic Ci to elevate apoptosis. Given *rdx* is a transcriptional target of Hh signaling, we proposed that the Hh pathway accelerates apoptosis through Rdx. In addition, we demonstrated that Rdx bound Diap1 promotes K63-linked polyubiquitination of Diap1, without affecting Diap1 stability. Finally, we revealed Rdx suppressed Diap1−Dronc interaction. Taken together, our findings uncover a Hh-Ci-Rdx axis promotes apoptosis through inhibiting Diap1-mediated Dronc degradation.

In *Drosophila*, forced expression of positive components of the Hh pathway in the wing disc will induce overgrowth [33], indicating its ability to promote cell proliferation. However, the function of the Hh pathway in apoptosis is still unclear. Although it reported that the mammalian Hh pathway suppresses apoptosis via activating anti-apoptotic gene *Bcl2* in tumor cells, no evidence supports the Hh pathway is able to activate *Bcl2* orthologs in *Drosophila*. In addition, Buffy and Debcl, two counterparts of Bcl2 in *Drosophila*, do not play a key role in apoptosis. In this study, we provided enough data to support that the Hh pathway promotes apoptosis through Rdx. Although Rdx is sufficient to trigger apoptosis, overexpression of *ci* does not decrease the wing disc size, possibly due to Ci activating pro-proliferative genes expression to mask Rdx’s effect. Consistently, overexpression of *ci* using *GMR-gal4* does not decrease the wild-type eye [33], suggesting that the Hh pathway plays its pro-apoptotic under *GMR*-*hid* background. It will be interesting to test whether the Hh pathway promotes apoptosis in *rpr*- and *grim*-overexpressing backgrounds.

Rdx binds two SBCs of Diap1 through its N-terminal MATH domain, indicating Diap1 is a possible substrate of Rdx E3 ligase. The following biochemical assays confirm that Rdx promotes K63-linked ubiquitination of Diap1. Rdx does not affect Diap1 protein level, showing that Rdx regulates Diap1 in a degradation-independent manner. The previous study has shown that Rdx prefers to add K48-linked Ub chains on Ci, leading to proteasome-mediated Ci degradation [48]. These results indicate that Rdx could add distinct modes of Ub chains on substrates to achieve different regulations. Supportively, Rdx’s mammalian ortholog SPOP targets inverted formin2 (INF2) for polyubiquitination to control its subcellular localization, without influencing its degradation [49].

Human Spop protein shares about 80% amino acid sequence identity with *Drosophila* Rdx, and they show a high degree of functional similarity [33]. *Drosophila* phenotypes caused by Rdx deficiency could be rescued by Spop expression [33]. Recently, Spop has garnered more attention due to its important role in tumorigenesis. Many studies have shown that Spop is frequently mutated in several types of tumors, such as prostate cancer and glioma [50, 51]. Resisting cell death is one of the hallmarks of cancer [52]. It will be fruitful to test whether Spop regulates tumorigenesis through promoting apoptosis. Intriguingly, the exome sequencing using 112 human prostate tumor samples shows that most mutations of Spop localize on its MATH domain [51]. A possible explanation is the MATH domain mutation attenuates its interaction with substrates, including IAPs, to relieve the pro-apoptotic function of Spop in tumor cells.

## Materials and methods

### Fly stocks

Some stocks used in this study were kindly from Dr. Qing Zhang’s lab, including UAS-*rdx*-RNAi [33], UAS-HA-*rdx* [33], *rdx*-lacZ [33], UAS-Ub-K48R [32], UAS-Ub-K63R [32], UAS-Ub-K0 [32] and UAS-HA-*rdx*-Δ3box [32]. UAS-*ci* and *rdx*^*Δ6*^ were gifted from Junzheng Zhang’s lab. UAS-*ci*-RNAi (NIG #2125R-1), UAS-P35 (BDSC #5072), UAS-*smo* (BDSC #44620), *GMR*-*hid* (BDSC #5771), *Ay-gal4* (BDSC #4411), UAS-GFP (BDSC #1522), *GMR-gal4* (BDSC #8605), MS1096 (BDSC #8860), UAS-Diap1 (BDSC #6657), *Ap*-*gal4* (BDSC #3041) were obtained from NIG or BDSC. The detailed information of these fly stocks has been described in Flybase database. The *tub*-Myc-*diap1* construct was made by cloning a full-length *diap1* cDNA downstream of the *α-tubulin* promoter [53]. Then the *tub*-Myc-*diap1* construct was injected into *w*^*1118*^ *Drosophila* embryos according to the method described previously [21].

### DNA constructs

To generate Myc-Diap1, Flag-Diap1, Flag-Rdx, Myc-Rdx, HA-Ub, and Myc-Dronc constructs, we amplified the corresponding cDNA fragments using Vazyme DNA polymerase (P505), and inserted them into pcDNA3.1-Myc, pCMV-Flag, or pCMV-HA backbone vectors respectively. Truncated constructs including Myc-Rdx-MATH (aa1-179), Myc-Rdx-BTB (aa180-374), and Flag-Rdx-Δ3box (deletion aa299-330) were made by inserting the corresponding coding sequences into pcDNA3.1-Myc or pCMV-Flag vectors. Flag-Diap1-M1, Flag-Diap1-M2, Flag-Diap1-M1/2, HA-Ub-K0, HA-Ub-K48, HA-Ub-K63, HA-Ub-K6R, HA-Ub-K11R, HA-Ub-K27R, HA-Ub-K29R, HA-Ub-K33R, HA-Ub-K48R, and HA-Ub-K63R were made by PCR-based site-directed mutagenesis.

### Immunostaining and confocal

Immunostaining of wing and eye disks was carried out according to our previous protocols [54]. Briefly, third-instar larvae were dissected in PBS and fixed in freshly made 4% formaldehyde in PBS at room temperature for 20 min, then washed three times with PBT (PBS supplemented with 0.1% Triton X-100). Larvae were incubated overnight with primary antibodies in PBT at 4 °C, then washed with PBT for three times and incubated with fluorophore-conjugated secondary antibodies for 2 h at room temperature. After washed for three times in PBT, disks were separated and mounted with 40% glycerol. Images were captured with Zeiss confocal microscope. Primary antibodies used in this study were shown as follows: rabbit anti-PH3 (1:100, Abcam); rat anti-Ci (1:50, DSHB); rabbit anti-Cas3 (1:100, ABclonal); mouse anti-β Gal (1:500, Santa Cruz); mouse anti-HA (1:200, Santa Cruz); mouse anti-Myc (1:200, Santa Cruz); rabbit anti-DrICE (1:100, CST) and rabbit anti-DIAP1 (1:100) [55]. All secondary antibodies used in this study were bought from Jackson ImmunoResearch, and were diluted at 1:500.

### Cell culture, transfection, and immunoblot

All cell-based assays in this study were carried out in 293T cells. 293T cells were cultured in Dulbecco’s modified Eagle’s medium (Gibco) supplemented with 10% fetal bovine serum (FBS, Gibco) and 1% penicillin/streptomycin (Sangon Biotech). Construct transfection was performed using PEI (Sigma) according to the manufacturer’s instructions [54]. Two days after transfection, cells were harvested to extract total protein for following co-IP and immunoblot (IB) according to our previous protocols [55]. The following antibodies were used for IP and IB: mouse anti-Flag (1:500 for IP, 1:5000 for IB, Sigma); mouse anti-Myc (1:200 for IP, 1:2000 for IB, Santa Cruz); mouse anti-HA (1:200 for IB); mouse anti-Actin (1:5000, Genscript); rabbit anti-Ub-K63 (1:1000 for IB, ABclonal); rabbit anti-Ub-K48 (1:1000 for IB, ABclonal); goat anti-mouse HRP (1:10000, Abmax) and goat anti-rabbit HRP (1:10,000, Abmax). The densities of IB bands were measured by Image J software.

### Eye size quantification and statistical analysis

For eye size analysis, all groups were crossed at 25 °C. Photos of adult eyes were taken on female flies at the same magnification. Sizes of eyes were measured by Image J software. Statistical analyses were performed with GraphPad Prism software, using one-way ANOVA. All data were presented as means ± standard deviation (SD), and *P* < 0.05 was considered statistically significant. Where exact *P*-values are not shown, statistical significance is shown as with *P < 0.05, **P < 0.01, ****P < 0.0001, and ns no significance.

## Data Availability

The datasets used and analyzed in this study are available from the corresponding author on reasonable request.
